# Double calcification in PLNTY: an unusual presentation of a rare tumor in young patients

**DOI:** 10.1055/s-0045-1809933

**Published:** 2025-07-28

**Authors:** Diego Jordão Lino Dias, Camilla Akemi Felizardo Yamada, Carmen Lucia Penteado Lancellotti, Lázaro Luíz Faria do Amaral

**Affiliations:** 1 Hospital Beneficência Portuguesa de São Paulo, Divisão de Neurorradiologia, São Paulo SP, Brazil; 2 Hospital Beneficência Portuguesa de São Paulo, Divisão de Oncologia Clínica, São Paulo SP, Brazil.; 3 Santa Casa de São Paulo, Faculdade de Ciências Médicas, São Paulo SP, Brazil.

**Figure 1:**
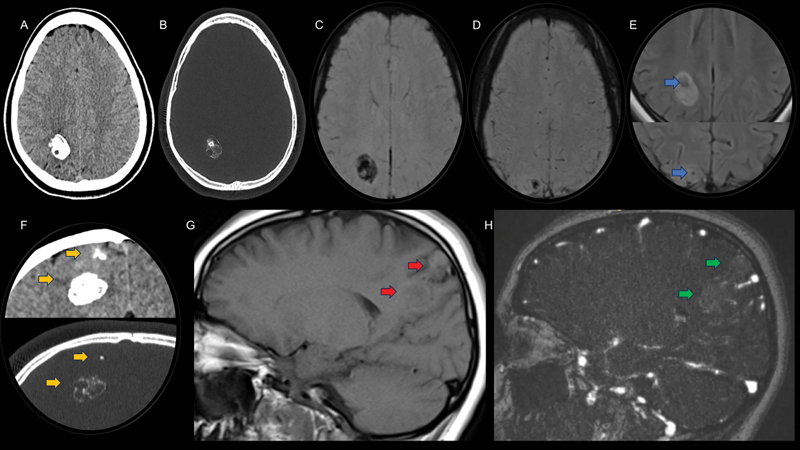
Head computed tomography (CT) scan ( **A,B** ) demonstrating a deep, solid, and heterogeneously-calcified parenchymal lesion in the right parietal lobe. Axial susceptibility-weighted imaging (SWI) magnietic resonance imaging (MRI) scan ( **C,D** ) showing marked hypointensity due to calcification, with emphasis on a smaller component of this lesion in the cortico-subcortical region of the right superior parietal lobule. Axial fluid-attenuated inversion recovery (FLAIR) MRI scan ( **E** ) demonstrating the lesion at two levels, with a predominance of hyperintense signal and hypointense areas in the more densely-calcified regions (blue arrows). A Head CT scan ( **F** ), presented in soft tissue and bone windows on the same coronal plane, better characterizes the dual calcification pattern (yellow arrows). Sagittal T1-weighted MRI scan ( **G** ) showing the lesion's extension from the cortico-subcortical region to the deep white matter, surrounded by a slight hyperintense halo (red arrows). Venous MR angiography ( **H** ) revealing mild enhancement of the lesion with paramagnetic contrast (green arrows).

**Figure 2:**
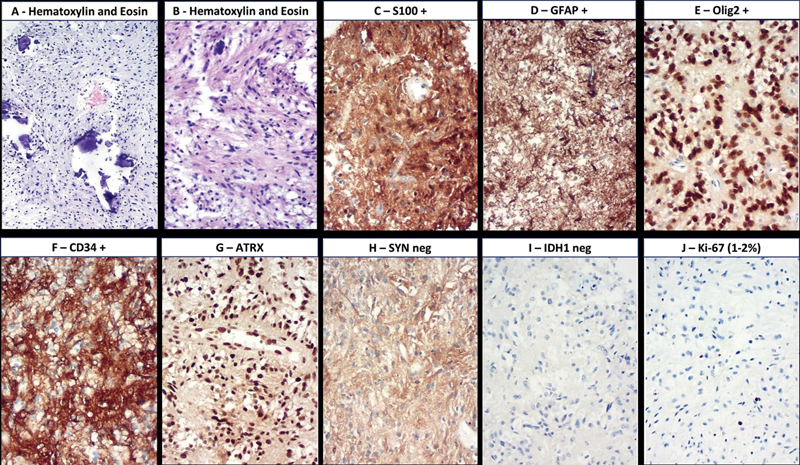
( **A** ) Hematoxylin and eosin staining (H&E) showing small, round, oligodendroglioma-like cells with slight nuclear variability. ( **B** ) H&E staining revealing dense calcifications within both adjacent tumor and non-tumor components. ( **C** ) S100-positive cells, ( **D** ) GFAP-positive cells, ( **E,F** ) Olig2-positive cells, and ( **F** ) CD34-positive cells are an important finding to distinguish from oligodendrogliomas. ( **G** ) Preserved ATRX. ( **H** ) SYN-negative. ( **I** ) IDH-1-negative. ( **J** ) Very low Ki-67 index (1–2%). *BRAF* V600E gene mutations were not found.

A 17-year-old, previously healthy, female patient presented with new-onset seizures. Computed tomography and magnetic resonance imaging scans ( Figure 1 ) revealed a solid lesion in the right parietal lobe with two densely calcified areas, suggestive of neoplastic characteristics, which was surgically resected for diagnostic clarification. The histopathological and immunohistochemical analyses ( Figure 2 ) confirmed the diagnosis of polymorphous low-grade neuroepithelial tumor of the young (PLNTY). Postoperatively, the patient improved clinically and remains disease-free.

The 2021 World Health Organization classification for PLNTY describes features such as oligodendroglioma-like cellular morphology, microcalcifications, low Ki-67 index, and absence of necrosis. 1 The imaging findings and the tumor's location in the right, non-dominant hemisphere, support the diagnosis. 2 3
